# A National Medical Benevolent Fund

**Published:** 1892-05

**Authors:** 


BUFFALO MEDICAL AND SURGICAL JOURNAL

A MONTHLY REVIEW OF MEDICINE AND SURGERY.

*EDITORS:*

THOMAS LOTHROP, M. D. - - WM. WARREN POTTER, M. D.

All communications, whether of a literary or business character, should be addressed to the managing editor: 284 Franklin Street, Buffalo, N. Y.

Vol. XXXI. MAY, 1892. No. 10.

### A NATIONAL MEDICAL BENEVOLENT FUND.

Dr. Frederick Horner, in an interesting paper published in the *Journal of the American Medical Association*, April 16, 1892, advocates the creation of an American Medical Benevolent Fund, the object of which shall be to afford immediate pecuniary relief to distressed qualified members of the medical profession, or their widows and orphans in case of death of a member. Dr. Horner cites the example of the British Medical Benevolent Fund, which shows that it is doing a most admirable work in the way of relieving those members who come within the range of its benevolence. There are also several States that have established physicians' aid associations, and these are referred to in Dr. Horner's paper. A number of cities or counties have also organized similar institutions, all of which are doing their work in the most satisfactory manner. We are of the opinion that it is wise for physicians to seek mutual protection of the kind that is indicated in Dr. Horner's admirable article, and hope that his paper will lead to an agitation of the subject, and a consequent establishment of the system.

The New York Mutual Aid Association, which is referred to in the paper, is doing a most gratifying work, and it seems to us there is no reasonable excuse why its membership should not be very much increased. In the State of Kentucky such an association numbers over 2,000 members, and it would seem as though New York should easily have an association of 4,000 or 5,000 physicians. Let Buffalo do her share, at any rate, and then we shall not feel ashamed of our attitude toward this good cause. There is no good or logical argument why physicians should not protect themselves, in the manner indicated, while there are many in its favor.

